# Volume Regulation and Renal Function at High Altitude across Gender

**DOI:** 10.1371/journal.pone.0118730

**Published:** 2015-03-05

**Authors:** Bernd Haditsch, Andreas Roessler, Peter Krisper, Herwig Frisch, Helmut G. Hinghofer-Szalkay, Nandu Goswami

**Affiliations:** 1 Institute of Physiology, Center of Physiological Medicine, Medical University of Graz, Harrachgasse 21, 8010, Graz, Austria; 2 Department of Internal Medicine, Division of Nephrology, Medical University of Graz, Auenbruggerplatz 27, 8036, Graz, Austria; 3 Department of Pediatrics, University Hospital Vienna, Waehringer Guertel 18-20, 1090, Vienna, Austria; University of Bari Aldo Moro, ITALY

## Abstract

**Aims:**

We investigated changes in volume regulating hormones and renal function at high altitudes and across gender.

**Methodology:**

Included in this study were 28 subjects (n = 20 males; n = 8 females. ages: 19 – 65 yrs), who ascended to a height of 3440m (HA1), on the 3^rd^ day and to 5050m (*HA2*), on the 14^th^ day. Plasma and urinary creatinine and urinary osmolality as well as plasma levels of plasma renin activity (PRA), Aldosterone, antidiuretic hormone (ADH), and atrial natriuretic peptide (ANP) were measured. The plasma volume loss (PVL) was estimated from plasma density and hematocrit. Glomerular filtration rate (GFR) was measured based on nocturnal (9 hour) creatinine clearance; this was compared with various methods for estimation of GFR.

**Results:**

The mean 24-hour urine production increased significantly in both sexes across the expedition. But PVL reached significance only in males. No changes in Na^+^ in plasma, urine or its fractional excretion were seen at both altitudes. Urinary osmolality decreased upon ascent to the higher altitudes. ADH and PRA decreased significantly at both altitudes in males but only at HA2 in females. However, no changes in aldosterone were seen across the sexes and at different altitudes. ANP increased significantly only in males during the expedition. GFR, derived from 9-h creatinine clearance (CreaCl), decreased in both sexes at HA1 but remained stable at HA2. Conventional Crea[p]-based GFR estimates (eGFR) showed only poor correlation to CreaCl.

**Conclusions:**

We report details of changes in hormonal patterns across high altitude sojourn. To our knowledge we are not aware of any study that has examined these hormones in same subjects and across gender during high altitude sojourn. Our results also suggest that depending on the estimation formula used, eGFR underestimated the observed decrease in renal function measured by CreaCl, thus opening the debate regarding the use of estimated glomerular filtration rates at high altitudes.

## Introduction

The kidneys play a crucial role in human adaption to high altitude, during acclimatization and in mountain sickness syndromes through their roles in regulating body fluids, electrolyte and acid–base homeostasis [1].

Besides hypoxia-driven erythropoietin-production and release, diuresis and natriuresis are well-known responses to acute hypoxic exposure. Since 1944 many *in vivo* and *in vitro* studies dealt with the so-called hypoxic diuretic response (HDR) [2–7]. HDR occurs between 16 and 10% of inspired oxygen or hypobaric equivalents [4–7], and is even independent of renal hemodynamics. Hypoxia itself appears to be the main stimulus of HDR, as isolated hypoxic perfusion of the carotid body causes hypoxic diuresis and natriuresis by inhibition of renal tubular sodium reabsorption [8]. In contrast, the diuretic effects of increased central venous or arterial pressure have been excluded as a cause for HDR [3, 7]. Hildebrandt et al. demonstrated a major contribution of systemic hypoxemia and hypocapnia to the early HDR, coming about without natriuresis and unrelated to O_2_ chemosensitivity [9]. It is usually assumed that full-blown HDR occurs after 24–72 hrs of hypoxic exposure. For instance, in laboratory-controlled conditions of up to 13 hours, no differences in urine output between simulated altitude (2800 m) and sea level have been seen [7].

As HDR is still increased in renal denervation, renal water and sodium excretion must involve additional regulation via hormones. Volume regulation is considered central both to high altitude adaptation and to maladaptive development of mountain sickness. However, the role of volume-regulating hormones in mediating HDR is still contentious. In field studies [4–6] as well as under laboratory conditions of acute hypoxic exposure [10] there was only poor correlation found between plasma levels of atrial natriuretic peptide (ANP), antidiuretic hormone (ADH), renin, aldosterone or urodilatin with diuresis or natriuresis. However, HDR appears to be influenced by several hormones, such as epinephrine and adrenomedullin [10]. On the other hand, some recent reported evidence has suggested that the rapid and powerful diuretic response to the hypobaric hypoxic stimulus of altitude is accompanied by decreased circulating concentrations of antidiuretic hormone, renin and aldosterone, increased levels of natriuretic hormones, plasma and urinary epinephrine, norepinephrine, endothelin and urinary adrenomedullin, with increased insensible fluid losses and reduced fluid intake [1]. Furthermore, the timing of altitude increases and adaptation also modifies the body’s physiologic responses to altitude [1].

Renal function (RF) has also been investigated under hypoxic conditions [11]. In clinical studies, the effects of acute or chronic hypoxia on renal function have been only reported in relation to ischemic circumstances or complications [12]. Furthermore, data on changes in glomerular filtration rate (GFR) at high altitude (HA) are very scarce. Therefore, in this study we determined plasma volume changes, urine osmolalities, fractional Na^+^ excretion and volume regulating hormonal changes in 28 subjects of both sexes during sojourn at high altitude for two weeks. In addition, we measured GFR based on creatinine clearance and assessed if the measured GFR was correlated with GFR calculated using the CKD-EPI (Chronic Kidney Disease Epidemiology Collaboration), the MDRD (Modification of diet in renal disease), the Cockcroft-Gault or the Mayo formula [CreaCl_CKD-EPI_, CreaCl_MDRD_, Crea Cl_CG_, Crea Cl_M_; [13–16].

## Methods

The study received approval from the Ethics Committee of the Medical University of Graz. Subjects gave both verbal and written consent. The Project *Silver Pyramid* was an interdisciplinary HA research expedition. It was carried out in the Khumbu Himal/Nepal for 4 weeks.

The subjects were informed about the possible risks and the experimental procedure. No subject had history of a recent or past renal impairment. After giving informed consent, the subjects underwent a medical, physical and mental examination. A baseline examination was performed at low altitude (LA, Herxheim, Germany, 150m), 6 weeks preceding the ascent. Between baseline examination and expedition, none of the subjects had a HA sojourn. During the field study in Nepal, each subject underwent 2 examinations, one during the adaptation on the third day of exposition to HA (Namche Bazar, 3440m, HA1) and another after two weeks acclimatization to HA at the so-called “silver pyramid” (Italian-Nepalesian research center Ev-K_2_-CNR, Lobuche, 5050m, HA2). During HA sojourn the subjects were checked for AMS, using the Lake Louise Consensus Group scale [17] as well as pulse-oximetry twice a day.

There was a relatively moderate daily physical exertion of 6–8 hrs, and the elevation profile was moderate as well. Investigation started 36 hrs after reaching HA1 / HA2. The previous day was used as relaxation period, without altitude change or any major physical effort.

Logistics had to be kept at a minimum level. Throughout the whole trip, food intake was not standardized and fluid intake was allowed *ad libitum*. There was no soda pop and coffee consumption and only some tea in the morning and at noon, orange juice in the evening. Food consisted of usual Sherpa meals; no food additives were provided. Special expedition doctors monitored any medication of the subjects. None of the subjects had any diuretic drug at HA1 and HA2 and the prior days.

At day 3 and 14, fasting antecubital venous blood samples were taken between 6.30 and 7.30 a.m. in a sitting position after a ≥15 min resting period. The blood was centrifuged immediately, and the plasma was kept frozen (at −20°C during the expedition; at −80°C until measurements in our laboratory in Graz were done).

As we anticipated stable conditions during the overnight sleeping periods (absence of any physical exertion, no fluid intake and narrow range of ambient temperature), and the urine samples could not have been stored, urine was collected between 10.00 p.m. the day preceding blood sampling and at 7.00 am (overnight urine collected over 9 hours). Urine volumes were determined, an aliquot taken and stored at −20/−80°C. In addition, urine made over the day was also collected to obtain 24-hour urinary volume. Proper functioning of the mobile deep-freezer was permanently controlled by using a minimum-maximum-thermometer.

Plasma and urinary creatinine (Crea[p], Crea [u]) were determined with an automatized analyser (Cobas Mira, Roche Inc., Switzerland). Urinary and plasma sodium was determined with an electron-sensitive device (AVL 988-4, AVL, Graz, Austria). Urine osmolalities were measured with a freezing point depression osmometer (Fisk One-Ten, Fiske Associates, Uxbridge, MA).

Fractional Na excretion was calculated by the following formula:

$$Fe_{\left(Na\right)}=\left(Sodium_{\left(Urine\right)}*Creatinine_{\left(Serum\right)}\right)/\left(Sodium_{\left(Serum\right)}*Creatinine_{\left(Urine\right)}\right)*100$$

### Hormonal measurements

After centrifugation of blood samples at 2500 rpm at 4°C for 15 minutes, plasma was decanted and stored at −20°C in Trasylol (aprotinin, 500 kallikrein inhibition units per ml) prechilled tubes and kept frozen until measurement. Aldosterone was measured using a competition assay (Immunotech RIA kit IM1664), plasma renin activity (PRA) with an angiotensin I-coated tube 125I RIA (DiaSorin) and expressed as ng angiotensin-I formed per ml of plasma per hour of incubation (ng/ml/h) and antidiuretic hormone (ADH) using RIA kits (Stillwater, MN, USA) after prior extraction (detailed in [18–19]). Hormone levels were above detection limit in all samples.

### Hematocrit, plasma density

Plasma albumin and total protein content were measured and they were used along with hematocrit values to calculate the plasma volume losses (see [20]). Hematocrit (Hct) was determined in duplicate (10 min at 10,000 rpm). Plasma density (PD) was measured with a high-precision mass densitometry device (model 602 M, Paar KG, Graz, Austria) on 0.2 ml samples employing the mechanical oscillator technique. Density determinations were performed at 37.00 ± 0.02°C controlled by an ultrathermostat (Hetofrig, Denmark). Mass density (FD) of the fluid shifts were calculated from corresponding PD and Hct values as follows (see [20])

$$FD=PD_{SL}-\frac{Htc_{SL}(1-Htc_{HA})}{Htc_{HA}-Htc_{SL}}(PD_{HA}-PD_{SL})\left(g/l\right)$$ 1

Where SL = Sea level, and HA = high altitude state, respectively.

With known FD, the volume (FV) of fluid-shifts can be computed from plasma density changes. Fluid shifts were expressed as relative changes (%) of plasma volume when compared to sea level. With hemoconcentration (plasma volume loss) the following formula applies:

$$FV=100*\frac{PD_{SL}-PD_{HA}}{PD_{HA}-FD}PV_{SL}\left(\%PVSL\right)$$ 2

### GFR determinations

We measured GFR based on absolute creatinine clearance. In addition, we compared the measured GFR with estimated GFR (eGFR), which was calculated using the CKD-EPI (Chronic Kidney Disease Epidemiology Collaboration), the MDRD (Modification of diet in renal disease), the Cockcroft-Gault or die Mayo formula [CreaCl_CKD-EPI_, CreaCl_MDRD_, Crea Cl_CG_, Crea Cl_M_; [13–16].

### Data analysis

Data are presented as mean ± SD. A nonparametric Kruskal-Wallis test was used to compare the related conditions SL and HA1 (*), SL and HA2 (*) and HA1 and HA2 (#). As post-hoc test a subsequent Dunn test was applied for multiple comparisons of differences between the three conditions (Graph Pad, Prism 5.0). Differences were considered significant if *p*<0.05 for the null hypothesis.

## Results

33 subjects started the expedition. All group members reached HA1. At day 8 of HA sojourn, five members had to return because of increasing AMS symptoms, so they were not examined at HA2. For data analysis reported here only 28 subjects (n = 20 males; n = 8 females) were used. The males had a mean height of 178.6±5.1cm, weight: 78.0± 7.0kg, and BMI was 24.5±2.3 and the females had mean height of 167.0±4.7cm, weight: 63.3±8.1kg and BMI was 22.7±2.4 investigated at sea level. The hemodynamic variables at baseline, at HA1 and at HA2 are shown in **Table 1**.

**Table 1:** Anthropometric measurements and resting hemodynamic variables at baseline, at HA1 and at HA2.

	Sea level		High altitude 1		High altitude 2	
	Men	Women	Men	Women	Men	Women
Weight	78.0±7.0	63.3±8.1	—	—	72.4±6.5	61.3±7.3
Height	178.6±5.1	167.0±4.7	—	—	—	—
HR	63.8±9.8	70.8±6.4	80.0±14.2*	83.3±8.3	85.5±14.8*	85.1±12.9
SYS	128.5±13.3	116.9±8.8	133.7±19.0	125.6±15.0	132.8±22.0	119.0±23.5
DIA	81.5±7.3	79.4±12.1	80.3±12.9	80.0±13.1	85.3±12.2	88.0±8.6
MAP	97.2±8.8	91.9±10.6	98.1±13.9	95.3±13.0	101.1±15.0	98.5±8.6

Throughout the ascent, there was approximately 5% plasma volume loss (PVL) in females in relation to baseline sea-level values. However, in males nearly 10% PVL occurred at HA1. At HA2, PVL was not statistically significant from the sea-level values (**Table 2**).

**Table 2:** Selected plasma parameters and volume regulating hormones at different altitudes across gender.

Plasma	Sea level		High altitude 1		High altitude 2	
	Men	Women	Men	Women	Men	Women
Total protein (g/l)	68.5±4.3	65.6±4.3	70.6±4.1	67.1±1.7	71.4±4.6	70.1±2.5*
Albumin (g/l)	50.1±2.9	46.4±2.7	50.7±3.0	47.3±3.2	51.6±3.6	49.6±1.4*
Creatinine (mg/dl)	1.4±0.2	1.2±0.1	1.5±0.2	1.4±0.1	1.5±0.2*	1.4±0.1*
Sodium_Serum_ (mmol/l)	146±2.9	145±1.8	147±3.8	145±3.5	147±3.9	144±2.3
ADH (pg/ml)	1.6±1.1	0.7±0.3	0.7±0.6*	0.5±0.3	0.5±0.5*	0.2±0.2*
PRA (ng AT I/mL.h)	2.7±1.4	1.8±1.1	1.4±0.7*	0.9±0.5	1.1±0.7*	0.7±0.6*
Aldosterone (pg/ml)	182±78	223±203	134±44	131±44	142±89	152±97
ANP (pg/ml)	8.7±4.2	11.3±3.6	13.3±5.4*	14.0±5.6	15.5±9.9*	24.2±12.6
Plasma density (g/l)	1022±1.3	1021±1.4	1022±1.2	1020±0.7	1021±1.4	1021±1.0
PVL (%)			9.6±11.1*	5.3±14.0	4.3±12	5.3±10.1

Values shown are mean ±SD.

* represents p<0.05 values vs SL

# represents p<0.05 when comparing high altitude 1 vs high altitude 2.

Legend: ADH: Antidiuretic hormone; PRA: Plasma renin activity; ANP: Atrial natriuretic peptide; PVL: Plasma volume loss

The mean 24-hour urine production increased significantly in both sexes as the expedition continued (**Table 3**).

**Table 3:** Urine parameters at different altitudes across gender.

Urine	Sea level		High altitude 1		High altitude 2	
	Men	Women	Men	Women	Men	Women
9hr amount (ml)	512±285	507±177	687±425	699±467	1051±377* #	936±431
24 hr amount (ml)	1533±656	1960±625	2729±1396*	3419±1186*	3089±866*	4003±1183*
Creatinine (mg/9hrs)	941±254	611±112	710±230*	472±127	776±209	505±71
Sodium (mmol/9hrs)	52.1±28.5	43.4±21.3	50.0±19.2	45.7±31.0	91.1±30.9* #	68.2±28.7
Osmolality (mOsm/kg)	668±221	455±219	299±153*	251±207*	326±182*	215±72
FE_Na_ (%)	1.3±0.8	1.5±1.1	1.2±0.7	1.8±0.8	1.4±0.6	1.6±0.5

Values shown are mean ± SD.

* represents p<0.05 values vs SL

# represents p<0.05 when comparing high altitude 1 vs high altitude 2.

*Legend*: FE_Na_: Fractional Sodium excretion.

Compared to sea-level, at HA1 no changes in plasma Na^+^, urine Na^+^ or fractional Na^+^ excretion were seen (Table 2, Table 3). Compared to sea-level, at HA2 there were no changes in plasma Na^+^ in both sexes but there was a significantly higher absolute urinary Na^+^ content in males despite unchanged fractional Na^+^ excretion. In females at HA2 absolute urinary Na^+^ content and fractional Na excretion stayed stable throughout the study. Urinary osmolality decreased upon ascent to the higher altitudes together with sodium concentration in urine.

Antidiuretic hormone (ADH) and PRA decreased significantly at both altitudes in males but only at HA2 in females (Table 2). However, no changes in aldosterone were seen across the sexes and at different altitudes. Atrial natriuretic peptide (ANP) increased significantly only in males during the expedition.

Mean 24-hour urine production increased significantly in both sexes and this was accompanied by body weight loss (Table 1) and hemoconcentration. Compared to SL, total protein as a marker of hemoconcentration increased during the whole sojourn. Nocturnal diuresis was significantly enhanced at HA2, whereas at HA1 there was only a slight increase. Overnight urinary osmolality decreased at HA1 and HA2.

The GFR, derived from 9-h-absolute renal creatinine clearance (CreaCl), decreased in both sexes at HA1 but remained stable at HA2. We used standard formulae for calculating GFR, which do not require the inclusion of hemoconcentration. Furthermore, recalculation of GFR, taking into account relative plasma volume loss during high altitude sojourn, using measurements of hematocrit, total protein and plasma density, showed similar results.

Only poor correlation was found between the measured creatinine clearance (CreaCl) and estimated GFR by using the CKD-EPI, MDRD, the Cockcroft-Gault or die Mayo formula (eGFR_CKD-EPI._ eGFR_MDRD_. eGFR_CG_. eGFR_M_, **Table 4**), which all underestimated the decrease of GFR.

**Table 4:** Comparing absolute 9-h-creatinine clearance (CreaCl) with estimated values of the GFR using formulae postulated by Cockcroft-Gault across gender.

GFR	Sea level		High altitude 1		High altitude 2	
	Men	Women	Men	Women	Men	Women
Creatinine/9hrs (ml/min)	113±31	93±15	78±24*	62±19*	81±19*	67±9*
Creatinine/9hrs (ml/min/1.73m^2^)	65±17	54±8	45±14*	36±10*	42±17*	38±5*
Cockroft-Gault (ml/min)	78±15	63±10	72±15	55±14	69±12	54±9
MDRD (ml/min/1.73m^2^)	63±11	53±6	57±10	45±8	54±8*	44±4*
Mayo (ml/min/1.73m^2^)	78±17	76±10	69±17	61±15	64±14*	60±7*
CKD-EPI (ml/min)	78±16	65±14	72±15*	55±14	69±12	55±10

MDRD. Mayo and CKD-EPI in 28 subjects.

Values shown are mean ± SD.

* represents p<0.05 values vs SL

# represents p<0.05 when comparing high altitude 1 vs high altitude 2.

When comparing the subjects who reached HA2 with subjects who developed AMS-symptoms and consequently had to return to base there were no significant differences either in Crea[p] nor in Crea[u], diuresis or natriuresis values at HA1 (data not shown).

## Discussion

Our data suggest that the hemodynamic variables remain stable during both HA levels (Table **1**). The heart rate, however, increases significantly in men, which may be attributed to the greater plasma volume losses in men (10% in men). Furthermore, our study of plasma and urine changes at high altitude sojourn across the sexes shows that mean 24-hour urine production increased significantly in both sexes as the expedition continued, but plasma volume losses reached significance only in males (Table**s 2, 3**). With respect to the volume regulating hormones, ADH and PRA decreased significantly at both altitudes in males but only at higher altitudes (HA 2) in females. No significant changes in aldosterone were seen across the sexes and at different altitudes; however aldosterone showed a tendency towards decreases. In addition, ANP increased significantly only in males during the expedition. No changes in Na^+^ in plasma, urine or its fractional excretion were seen at both altitudes, suggesting that proper volume and water regulation was maintained. Urinary osmolality decreased upon ascent to the higher altitudes, presumably due to higher fluid intake. Finally, the GFR, derived from 9-h creatinine clearance (CreaCl), decreased in both sexes already at lower altitudes, no further deterioration was seen at higher altitudes (Table **4**). Our results also suggest that conventional Crea[p]-based GFR estimates (eGFR) show only poor correlation to CreaCl.

### Water balance and electrolytes

Water balance is often affected during high altitude sojourn. After several days of hypoxemia, total body water can be reduced by 1–3 liters, either due to a greater loss of insensible water because of lower humidity and increased respiration or due to a reduced intake of water and sodium because of poor thirst and appetite [21–22]). Maintenance of water intake at altitude, however, does not prevent the decrease in total body water, indicating that renal mechanisms are probably involved [21–22]. In addition, exposure to high altitude hypoxia increases also sodium and water diuresis, bringing about depletion of circulating volume and in consequence a relative increase in hematocrit to counterbalance the reduced supply of oxygen. Indeed during acute hypoxemia, renal excretion rates of sodium and water usually increases [9, 23], but thereafter stabilize at an unchanged or lower level compared with normoxemia [24]. Our data obtained during high altitude sojourn also show that urine concentration was reduced in hypoxia (Table **3**).

### Volume regulating hormones

Although differences in experimental conditions probably explain the contradictory data in literature on the hormonal control of sodium and water balance at HA, most of the studies available show either no change [4–5] or a decrease in sodium retaining hormones, particularly renin-angiotensin-aldosterone system (RAAS) [25–26]. Further, ANP levels have been found to be unchanged or increased, mainly in subjects with acute mountain sickness [5, 27–28]. Our data also show that PRA and aldosterone levels were constantly suppressed at both HA levels in both the sexes, whereas the ANP response was stimulated during acute HA exposure in both the sexes but reached significance only in males. This suggests that hypoxia-induced chemoreceptor stimulation may cause the natriuretic phenomenon through direct suppression of the RAAS [29].

Our data show that ADH decreases during ascent and is accompanied by diuresis, and decreased urinary osmolality in both sexes. This is in agreement with what has been reported [30].

As has been previously reported, ANP increases upon ascent to higher altitudes [5] and together with depressed ADH, could explain the diuresis occurring at high altitudes. However, natriuresis might not be a consequence of this ANP increase during hypoxia as the fractional Na excretion is unchanged (see Table **3**).

### Renal function changes measurements and assessments

We observed a roughly 30% fall of GFR measured by 9-hr creatinine clearance. The exact mechanism behind this substantial decrease remains speculative. Hypoxia per se or HDR related sympathetic activation [4–6] might play a pathogenic role.

It is interesting that various formulas for eGFR determination underestimated the “true” extent of GFR reduction. A diminished creatinine generation in high altitude, reflected by the decreased creatinine appearance in timed urine samples in our study, is a likely explanation. Lower generation results in lower serum creatinine at a given level of GFR. This leads to overestimation by eGFR formulas at high altitudes, as they primarily depend on serum creatinine. Why creatinine generation was diminished, remains open for debate.

There have been several cross-sectional and longitudinal studies, describing a temporal sequence of kidney function changes with increasing altitude [16, 24, 31–33]. In addition, controversial results have been obtained when GFR at different degrees of hypoxia was estimated using inulin clearance [34], creatinine clearance [32] or serum creatinine [35]. A limitation of using exogenously administered clearance markers such as inulin or isotopes to assess renal function is that they cannot be used at high altitudes.

A study suggested a linear decrease of 3.1 ml min^-1^ 1.73 m^2^ eGFR per 1000 m increase in altitude [36]. Our data do not, however, confirm these results. We observed that renal function decreased significantly at HA1, but showed no further deterioration at HA2.

### Limitations

A crucial aspect of any renal study is the need to strictly control and measure dietary, salt and fluid intake. This was not done in this study due to logistical difficulties posed by this field study. However, as all of the subjects ate the same food and drank the same type of fluids, we believe that it could have influenced the results of the study only minimally.

Additionally, the baseline measurements were not done immediately before high altitude sojourn. Due to logistical constraints typical of a field study, the baseline measurements were done six weeks before high altitude sojourn.

Finally, another limitation of the study could be that the estimated plasma volume loss calculations depend on the assumption that red cells are constant in number. As high altitude is now known to affect the red blood cell counts, it is possible that the plasma volume losses reported here may not be reflective of the actual plasma volume changes that occur during high altitude ascent.

## Conclusions

We report details of changes in hormonal patterns across high altitude sojourn. To our knowledge we are not aware of any study that has examined these hormones in the same subjects and across gender during high altitude sojourn. Our results also suggest that depending on the estimation formula used, eGFR underestimated the observed decrease in renal function measured by CreaCl, thus opening the debate regarding the use of estimated glomerular filtration rates at high altitudes.
